# Retraction: SNHG3 promotes proliferation and invasion by regulating the miR-101/ZEB1 axis in breast cancer

**DOI:** 10.1039/d1ra90025k

**Published:** 2021-01-21

**Authors:** Laura Fisher

**Affiliations:** Royal Society of Chemistry Thomas Graham House, Science Park, Milton Road Cambridge CB4 0WF UK advances-rsc@rsc.org

## Abstract

Retraction of ‘SNHG3 promotes proliferation and invasion by regulating the miR-101/ZEB1 axis in breast cancer’ by Liang Chang *et al.*, *RSC Adv.*, 2018, **8**, 15229–15240, DOI: 10.1039/C8RA02090F.

The Royal Society of Chemistry hereby wholly retracts this *RSC Advances* article due to concerns with the reliability of the data. The images in the article were screened by an image integrity expert.

A section of the si-con image (blue cells only) in Fig. 2E is duplicated in the si-con image in Fig. 2F, but the images are presented at different magnifications and have been flipped vertically. These figures represent different experiments.

The authors were asked to provide the raw data for this article, but did not respond. Given the significance of the concerns about the validity of the data, and the lack of raw data, the findings presented in this paper are not reliable.

The authors have been informed but have not responded to any correspondence regarding the retraction.

Signed: Laura Fisher, Executive Editor, *RSC Advances*

Date: 7^th^ January 2021

## Supplementary Material

